# Supplementary material for: Fe-N system at high pressure reveals a compound featuring polymeric nitrogen chains
Source: Nat Commun. 2018 Jul 16;9:2756. doi: 10.1038/s41467-018-05143-2 (PMC6048061; doi:10.1038/s41467-018-05143-2)
Supplement: Supplementary file 1 — Supplementary Information [file 41467_2018_5143_MOESM1_ESM.pdf]

Fe-N system at high pressure reveals a compound featuring polymeric nitrogen chains

M. Bykov et al.

Supplementary Note 1: Selected examples of diffraction images

The following examples show typical diffraction images from laser-heated samples. As one can see from the Supplementary Figures 1-3, the diffraction peaks from different phases and grains are very well separated from each other on the single-crystal diffraction datasets.

The interpretation of such datasets by their radial integration to an intensity-2theta plot is much more complicated and ambiguous due to many overlapping peaks in the high-angle region (Supplementary Figures 4, 5).

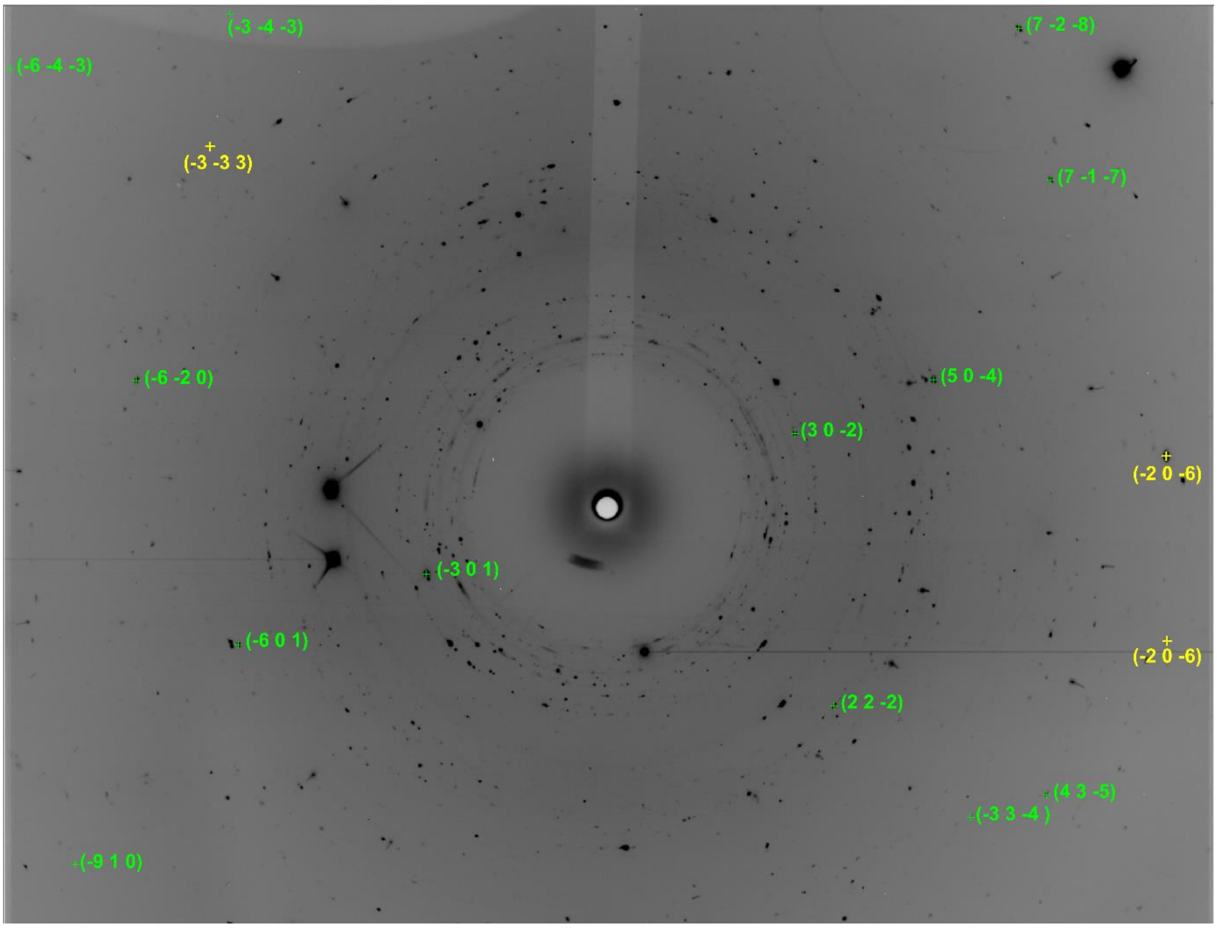

Supplementary Figure 1. One frame from the single-crystal X-ray diffraction dataset ($\Delta\omega$ = 0.5°) measured after laser-heating of Fe in N_2 at ~50 GPa. Reflections belonging to one grain of Fe_3N_2 are marked by green crosses. Reflections marked by yellow crosses belong to one grain of FeN. Non-indexed peaks are produced by the diffraction from different grains of the same phases, diamond, nitrogen and iron.

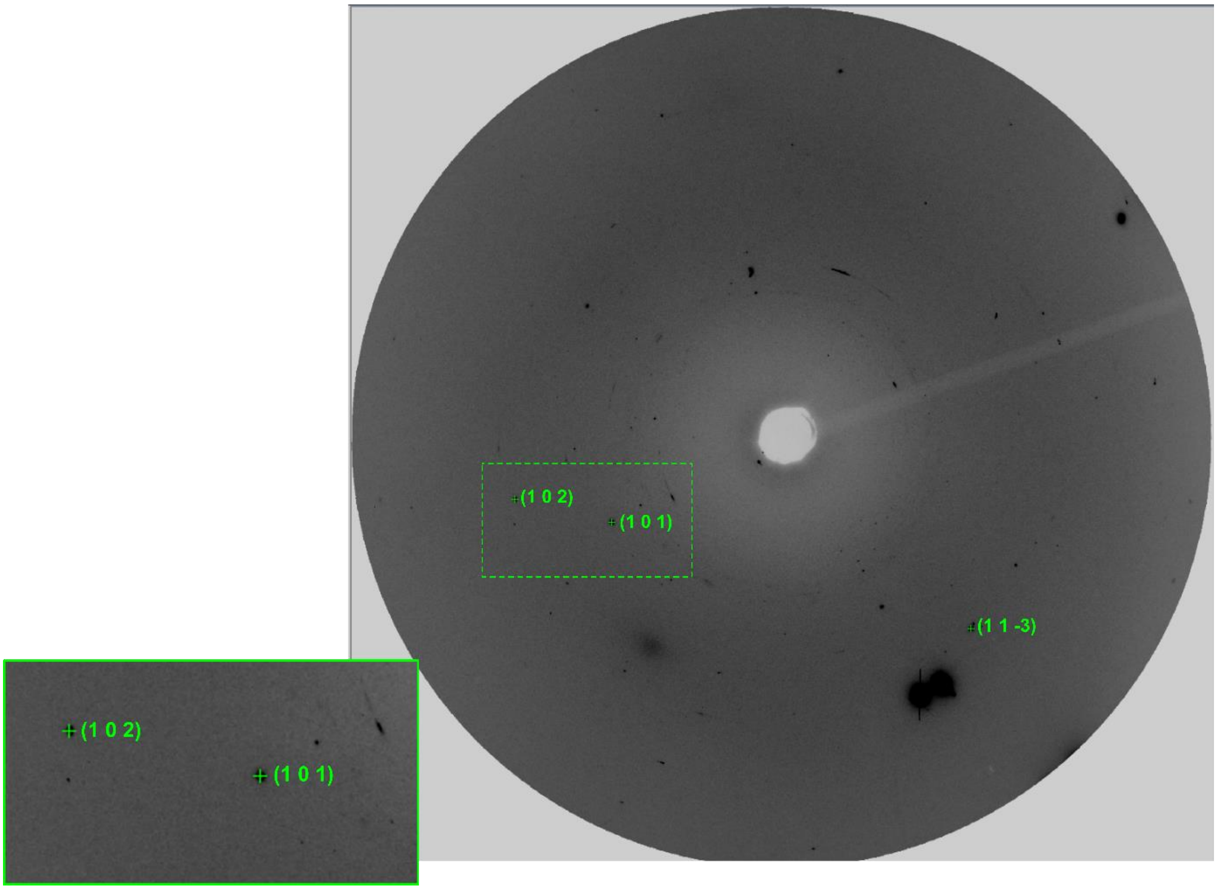

Supplementary Figure 2. One frame from the diffraction dataset at 135 GPa. Reflections belonging to one grain of FeN_4 are marked by green crosses. Non-indexed peaks are produced by the diffraction from different grains of the same phase, diamond and nitrogen.

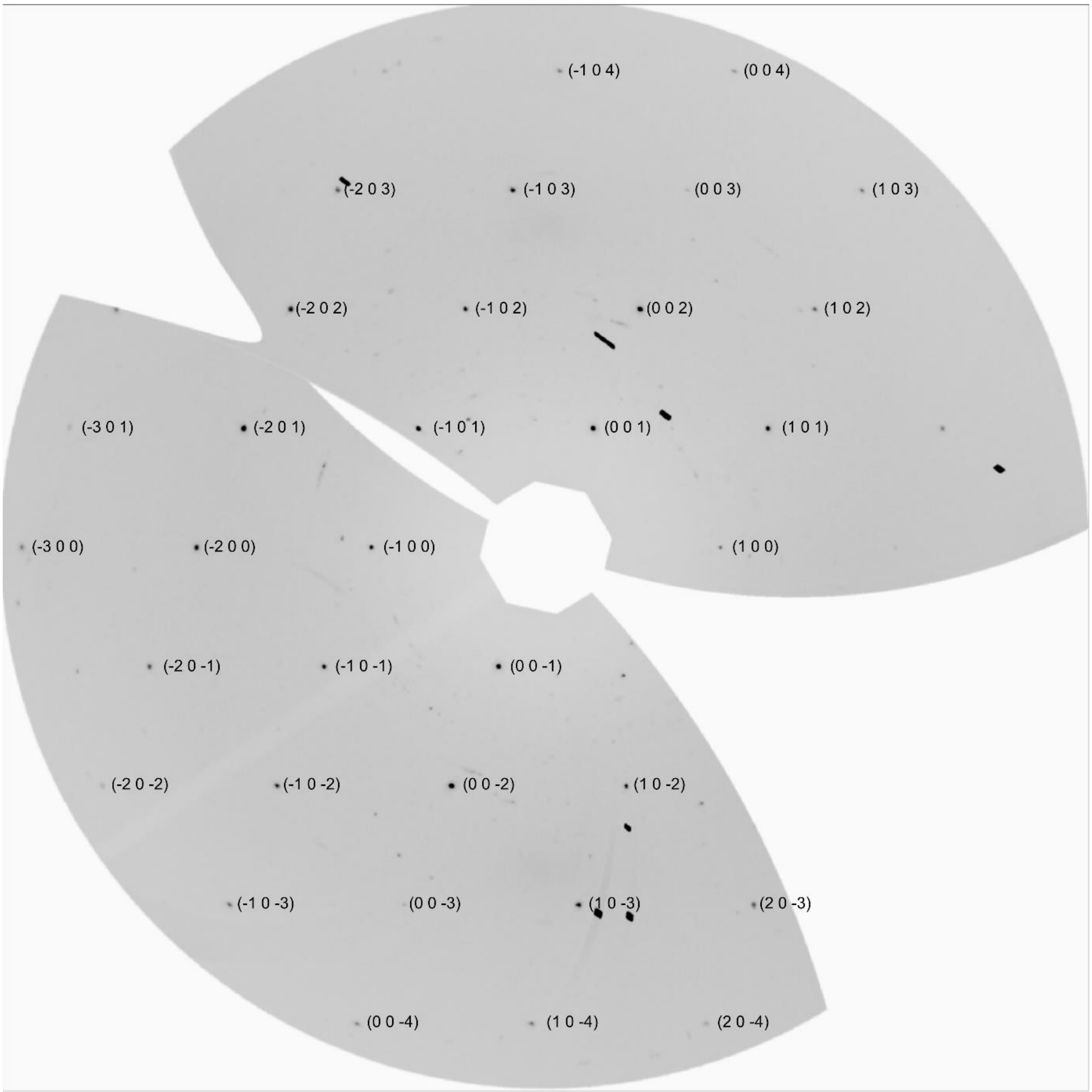

Supplementary Figure 3. Reconstructed reciprocal lattice plane (*h*0*l*) of FeN_4 at 135 GPa.

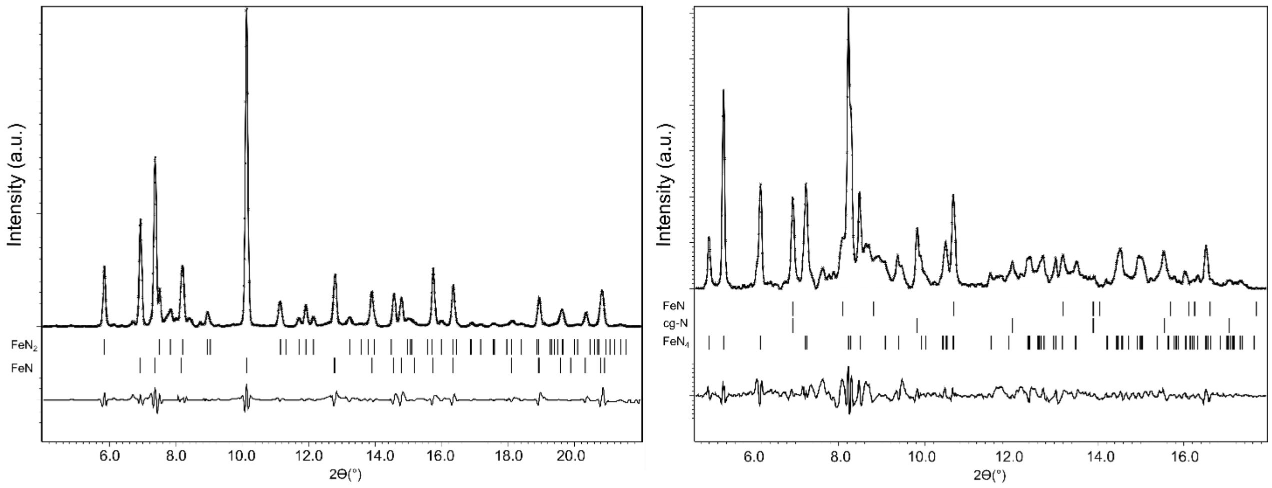

Supplementary Figure 4. Typical 2D-integrated diffraction patterns. Shown are the examples of a mixture of FeN and FeN_2 at 69.6 GPa (left) and FeN + FeN_4 at 135 GPa (right).

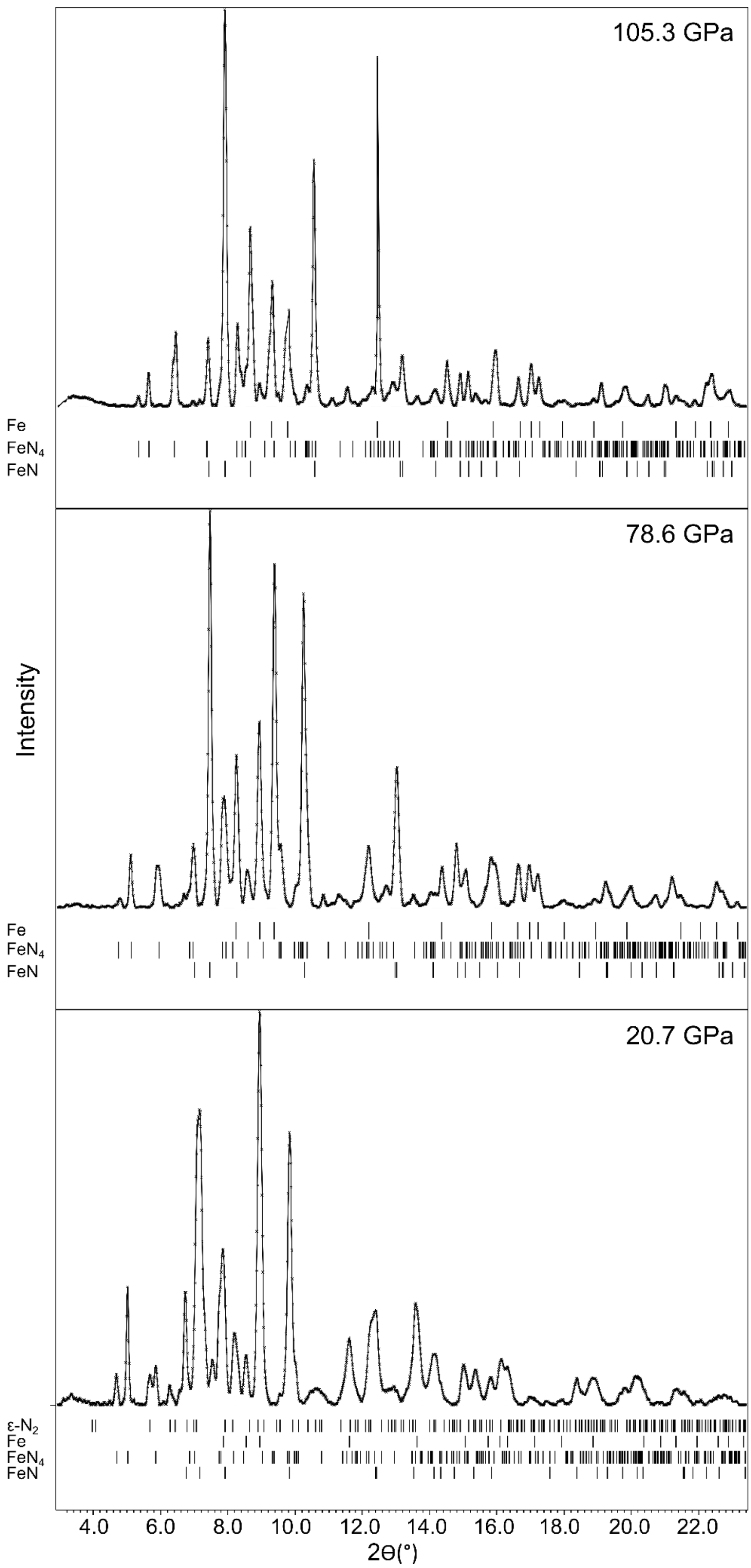

Supplementary Figure 5. 2D-integrated still images of the sample obtained in the experiment #3 showing the existence of the FeN_4 phase down to 22.7 GPa.

Supplementary Methods

Mössbauer spectroscopy

Synchrotron Mössbauer Source (SMS) spectra were recorded at the Nuclear Resonance Beamline ID18 of the ESRF [1] using the (111) nuclear reflection of a $^{57}FeBO_3$ single crystal mounted on a Wissel velocity transducer driven with a sinusoidal waveform[2]. The X-ray beam was focused down to 16x12 μm^2 (VxH) using Kirkpatrick-Baez mirrors. The linewidth of the SMS and the absolute position of the center shift relative to α-iron was controlled before and after each measurement using a $K_2Mg^{57}Fe(CN)_6$ reference single line absorber. The velocity scale was calibrated using 25 μm thick natural iron foil. The collection time of the spectrum presented in the supplementary Figure 6 was 10 hours. The spectrum was fitted using a transmission integral with a normalized Lorentzian-squared source line shape using the MossA software package[3].

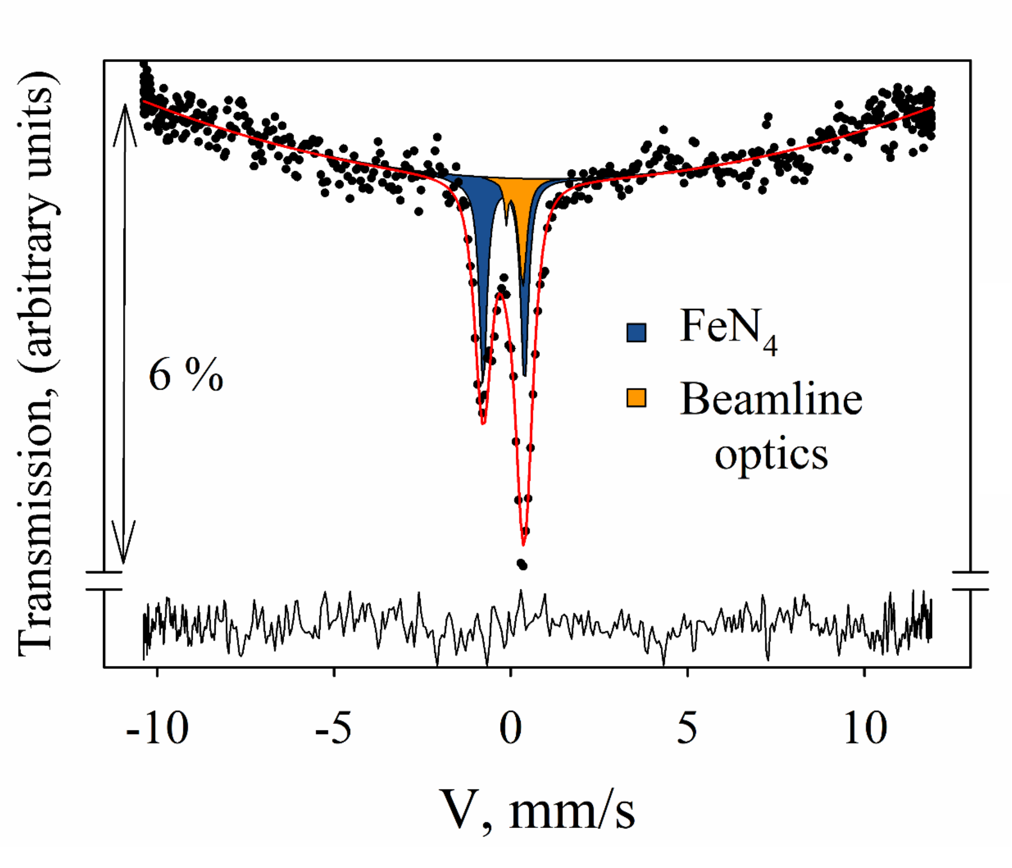

Supplementary Figure 6. Synchrotron Mössbauer Source spectrum of FeN_4 at ~135GPa and the room temperature. The FeN_4 contribution was fitted to a doublet (blue) with -0.199(14) mm/s center shift and 1.17(3) mm/s quadrupole splitting. The red solid line shows the theoretical fit and the residuals are indicated below the spectrum. The spectrum contains a contribution from iron impurities in the Be compound refractive lenses (orange) used to decrease the divergence of the beam incident to the Synchrotron Mössbauer Source.

Raman spectroscopy

The Raman spectra on the Fe-N_2 sample were collected using DilorXY and LabRam systems equipped with the He–Ne (the excitation wavelength 632.8 nm) laser source. The laser power 50 mW was applied. Raman spectra were collected in the region 200 – 3000 cm^{-1} by means of 2 accumulations for 60 seconds. The frequency resolution was 2 cm^{-1}.

The starting sample contains molecular nitrogen phase as evident from the vibron peaks around 2400 cm^{-1}. The laser-heating of the sample leads to the appearance of a small peak at ~ 843 cm^{-1} that perfectly corresponds to the peak of cg-N[4]. Further interpretation is not straightforward due to the strong fluorescence background.

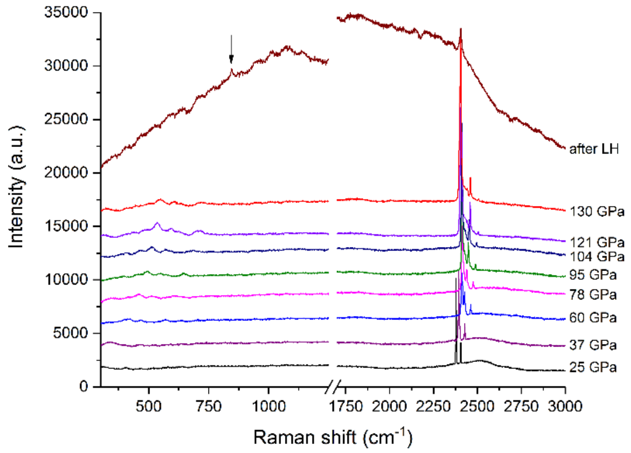

Supplementary Figure 7. Raman spectra of the Fe+N_2 sample on compression and after laser-heating. The region between 1250 and 1700 is skipped due to the strong Raman signal from the stressed diamond anvils. Arrow shows the peak of cg-N appearing after laser-heating.

Energy-density calculations

We have calculated gravimetric energy density (GED) and volumetric energy density (VED) of $P\bar{1}$ FeN_4 to estimate its practical applicability as promising high energy density materials. Calculated phonon dispersions and quasi-harmonic approximation (QHA) implemented in PHONOPY [5] have been applied to analyze the energy release of the reaction $FeN_4 \rightarrow FeN + (3/2)N_2$ at 300 K and P = 0 GPa. FeN was assumed in its anti-ferromagnetic (AFM) B8 structure type and the enthalpy and entropy of N_2 were derived from thermochemical tables[6] as suggested by Zhang et al. [7]. The free energy calculations were performed using the on-site screened Coulomb energy U=4.0 eV and the exchange energy J=1.0 eV for the Fe *d* states. The calculated chemical energy released by the reaction is 3.12 eV. In the framework of Debye-Grüneisen model one obtains 3.37 eV [8]. This agreement suggests the applicability of the simple Debye-Grüneisen approach. The 3.12 (3.37) eV energy corresponds to 2.68 (2.9) kJ/g gravimetric energy density or 12.61 (13.62) kJ/cm^3 volumetric energy density, where the volume of FeN_4 is 39.6 Å^3 at ambient pressure. The energy release of the reaction $FeN_4 \rightarrow Fe + 2N_2$ was estimated using the Debye-Grüneisen approach with ferromagnetic $Im\bar{3}m$ Fe. At ambient conditions this reaction results in 3.7 eV. This gives 3.2 kJ/g gravimetric or 15.03 kJ/cm^3 volumetric energy density.

Supplementary References:

1. Rüffer, R. & Chumakov, A. I. Nuclear Resonance Beamline at ESRF. *Hyperfine Interact.* **97–98,** 589–604 (1996).
2. Potapkin, V. *et al.* The ^{57}Fe Synchrotron Mössbauer Source at the ESRF. *J. Synchrotron Radiat.* **19,** 559–69 (2012).
3. Prescher, C., McCammon, C. & Dubrovinsky, L. MossA : a program for analyzing energy-domain Mössbauer spectra from conventional and synchrotron sources. *J. Appl. Crystallogr.* **45,** 329–331 (2012).
4. Eremets, M. I., Gavriliuk, A. G., Trojan, I. A., Dzivenko, D. A. & Boehler, R. Single-bonded cubic form of nitrogen. *Nat. Mater.* **3,** 558–63 (2004).
5. Togo, A. & Tanaka, I. First principles phonon calculations in materials science. *Scr. Mater.* **108,** 1–5 (2015).
6. *JANAF Thermo- chemical Tables*. (National Bureau of Standards, 1971).
7. Zhang, J., Oganov, A. R., Li, X. & Niu, H. Pressure-stabilized hafnium nitrides and their properties. *Phys. Rev. B* **95,** 20103 (2017).
8. Moruzzi, V. L., Janak, J. F. & Schwarz, K. Calculated thermal properties of metals. *Phys. Rev. B* **37,** 790–799 (1988).
