# Supplementary material for: Fe-N system at high pressure reveals a compound featuring polymeric nitrogen chains
Source: Nat Commun. 2018 Jul 16;9:2756. doi: 10.1038/s41467-018-05143-2 (PMC6048061; doi:10.1038/s41467-018-05143-2)
Supplement: Supplementary file 3 — Description of Additional Supplementary Files [file 41467_2018_5143_MOESM3_ESM.pdf]

Description of Additional Supplementary Files

File Name: Supplementary Data 1
Description: Crystallographic information file for Fe_3N_2 at 50 GPa.

File Name: Supplementary Data 2
Description: Crystallographic information file for FeN at 50 GPa.

File Name: Supplementary Data 3
Description: Crystallographic information file for FeN_2 at 58 GPa.

File Name: Supplementary Data 4
Description: Crystallographic information file for FeN_4 at 135 GPa.

File Name: Supplementary Data 5
Description: Checkcif report for Fe_3N_2 at 50 GPa.

File Name: Supplementary Data 6
Description: Checkcif report for FeN at 50 GPa.

File Name: Supplementary Data 7
Description: Checkcif report for for FeN_2 at 58 GPa.

File Name: Supplementary Data 8
Description: Checkcif report for FeN_4 at 135 GPa.

File Name: Supplementary Data 9
Description: *hkl* file for FeN_4 at 135 GPa.
