# Supplementary material for: Fe-N system at high pressure reveals a compound featuring polymeric nitrogen chains
Source: Nat Commun. 2018 Jul 16;9:2756. doi: 10.1038/s41467-018-05143-2 (PMC6048061; doi:10.1038/s41467-018-05143-2)

checkCIF/PLATON report

Structure factors have been supplied for datablock(s) FeN2_58GPa

THIS REPORT IS FOR GUIDANCE ONLY. IF USED AS PART OF A REVIEW PROCEDURE FOR PUBLICATION, IT SHOULD NOT REPLACE THE EXPERTISE OF AN EXPERIENCED CRYSTALLOGRAPHIC REFEREE.

No syntax errors found. CIF dictionary Interpreting this report

Datablock: FeN2_58GPa

```
Bond precision:  N- N = 0.0070 A          Wavelength=0.28880

Cell:            a=4.4308(19)     b=3.7218(12)     c=2.4213(18)
                 alpha=90         beta=90          gamma=90
Temperature:     293 K

                Calculated                 Reported
Volume          39.93(4)                   39.93(4)
Space group     P n n m                    P n n m
Hall group      -P 2 2n                    -P -2xabc;-
Moiety formula  Fe N2                      ?
Sum formula     Fe N2                      Fe1 N2
Mr              83.87                      83.90
Dx,g cm-3       6.976                      6.975
Z               2                          2
Mu (mm-1)       1.426                      1.322
F000            80.0                       80.0
F000'           80.16
h,k,lmax        9,7,4                      8,7,3
Nref            208                        109
Tmin,Tmax
Tmin'

Correction method= Not given

Data completeness= 0.524          Theta(max)= 17.280

R(reflections)= 0.0569( 68)       wR2(reflections)= 0.1157( 109)

S = 1.490                  Npar= 11
```

The following ALERTS were generated. Each ALERT has the format
test-name_ALERT_alert-type_alert-level.
Click on the hyperlinks for more details of the test.

Alert level A

```
PLAT029_ALERT_3_A _diffrn_measured_fraction_theta_full value Low .      0.660 Note
```

Alert level C

```
PLAT088_ALERT_3_C Poor Data / Parameter Ratio ....................       9.91 Note
PLAT127_ALERT_1_C Implicit Hall Symbol  Inconsistent with Explicit    -P -2xabc;-2ya
PLAT790_ALERT_4_C Centre of Gravity not Within Unit Cell: Resd.  #          1 Note
              Fe N2
PLAT910_ALERT_3_C Missing # of FCF Reflection(s) Below Theta(Min).          6 Note
PLAT911_ALERT_3_C Missing # FCF Refl Between THmin & STh/L=  0.600         11 Report
PLAT913_ALERT_3_C Missing # of Very Strong Reflections in FCF ....          4 Note
PLAT971_ALERT_2_C Check Calcd Residual Density  1.21A From       N       2.17 eA-3
PLAT971_ALERT_2_C Check Calcd Residual Density  1.31A From       N       2.02 eA-3
PLAT971_ALERT_2_C Check Calcd Residual Density  1.08A From      Fe       1.83 eA-3
PLAT971_ALERT_2_C Check Calcd Residual Density  0.70A From       N       1.59 eA-3
PLAT972_ALERT_2_C Check Calcd Residual Density  1.31A From       N      -2.24 eA-3
PLAT972_ALERT_2_C Check Calcd Residual Density  1.24A From       N      -2.12 eA-3
PLAT972_ALERT_2_C Check Calcd Residual Density  0.66A From      Fe      -1.92 eA-3
PLAT972_ALERT_2_C Check Calcd Residual Density  0.81A From       N      -1.86 eA-3
PLAT972_ALERT_2_C Check Calcd Residual Density  1.07A From       N      -1.53 eA-3
PLAT973_ALERT_2_C Check Calcd Positive Residual Density on      Fe       1.04 eA-3
PLAT975_ALERT_2_C Check Calcd Residual Density  0.73A From       N       1.31 eA-3
PLAT976_ALERT_2_C Check Calcd Residual Density  0.71A From       N      -1.35 eA-3
PLAT976_ALERT_2_C Check Calcd Residual Density  0.89A From       N      -1.05 eA-3
```

Alert level G

```
ABSMU01_ALERT_1_G  Calculation of _exptl_absorpt_correction_mu
                not performed for this radiation type.
PLAT004_ALERT_5_G Polymeric Structure Found with Maximum Dimension          3 Info
PLAT005_ALERT_5_G No Embedded Refinement Details found  in the CIF     Please Do !
PLAT199_ALERT_1_G Reported _cell_measurement_temperature ..... (K)        293 Check
PLAT200_ALERT_1_G Reported   _diffrn_ambient_temperature ..... (K)        293 Check
PLAT912_ALERT_4_G Missing # of FCF Reflections Above STh/L=  0.600         61 Note
```

---

1 **ALERT level A** = Most likely a serious problem - resolve or explain
0 **ALERT level B** = A potentially serious problem, consider carefully
19 **ALERT level C** = Check. Ensure it is not caused by an omission or oversight
6 **ALERT level G** = General information/check it is not something unexpected

4 ALERT type 1 CIF construction/syntax error, inconsistent or missing data
13 ALERT type 2 Indicator that the structure model may be wrong or deficient
5 ALERT type 3 Indicator that the structure quality may be low
2 ALERT type 4 Improvement, methodology, query or suggestion
2 ALERT type 5 Informative message, check

It is advisable to attempt to resolve as many as possible of the alerts in all categories. Often the minor alerts point to easily fixed oversights, errors and omissions in your CIF or refinement strategy, so attention to these fine details can be worthwhile. In order to resolve some of the more serious problems it may be necessary to carry out additional measurements or structure refinements. However, the purpose of your study may justify the reported deviations and the more serious of these should normally be commented upon in the discussion or experimental section of a paper or in the "special_details" fields of the CIF. checkCIF was carefully designed to identify outliers and unusual parameters, but every test has its limitations and alerts that are not important in a particular case may appear. Conversely, the absence of alerts does not guarantee there are no aspects of the results needing attention. It is up to the individual to critically assess their own results and, if necessary, seek expert advice.

Publication of your CIF in IUCr journals

A basic structural check has been run on your CIF. These basic checks will be run on all CIFs submitted for publication in IUCr journals (*Acta Crystallographica*, *Journal of Applied Crystallography*, *Journal of Synchrotron Radiation*); however, if you intend to submit to *Acta Crystallographica Section C* or *E* or *IUCrData*, you should make sure that full publication checks are run on the final version of your CIF prior to submission.

Publication of your CIF in other journals

Please refer to the *Notes for Authors* of the relevant journal for any special instructions relating to CIF submission.

PLATON version of 13/08/2017; check.def file version of 27/07/2017

Datablock FeN2_58GPa - ellipsoid plot

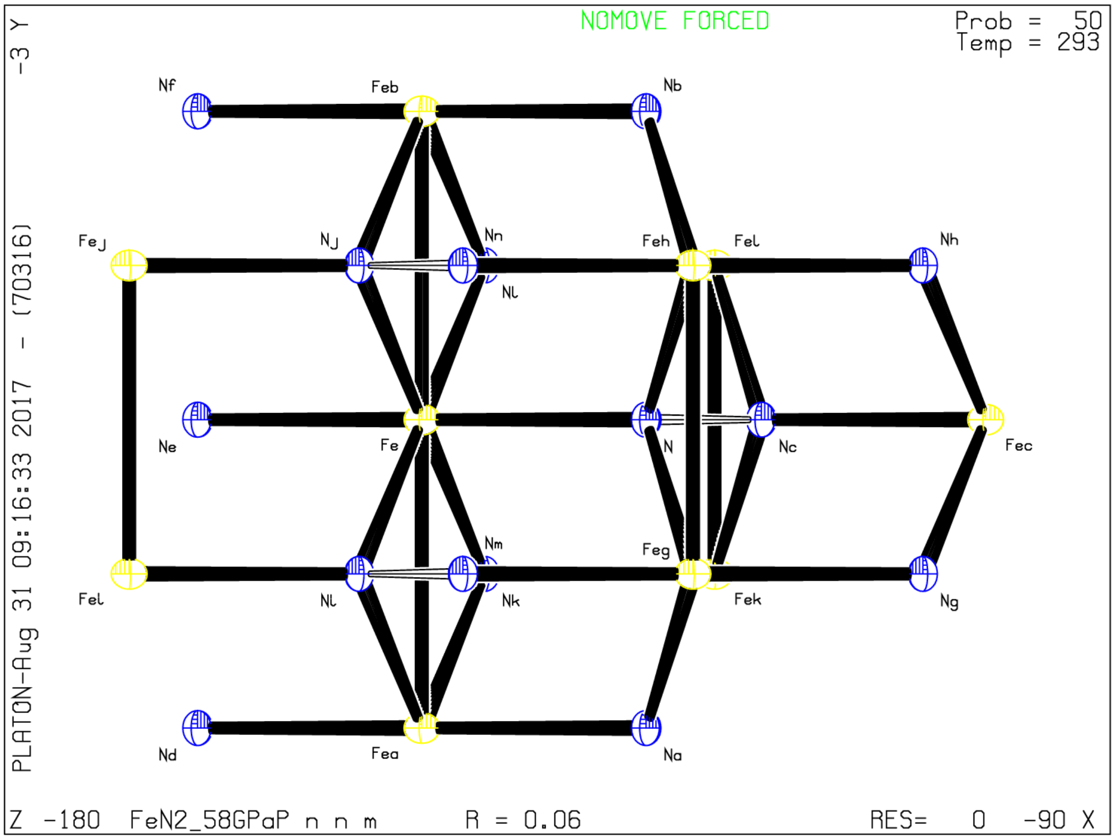

Supplement: Supplementary file 10 — Supplementary Data 7 [file 41467_2018_5143_MOESM10_ESM.pdf]
