# Supplementary material for: Fe-N system at high pressure reveals a compound featuring polymeric nitrogen chains
Source: Nat Commun. 2018 Jul 16;9:2756. doi: 10.1038/s41467-018-05143-2 (PMC6048061; doi:10.1038/s41467-018-05143-2)

checkCIF/PLATON report

Structure factors have been supplied for datablock(s) Fe3N2_49.6_GPa

THIS REPORT IS FOR GUIDANCE ONLY. IF USED AS PART OF A REVIEW PROCEDURE FOR PUBLICATION, IT SHOULD NOT REPLACE THE EXPERTISE OF AN EXPERIENCED CRYSTALLOGRAPHIC REFEREE.

No syntax errors found.   CIF dictionary   Interpreting this report

Datablock: Fe3N2_49.6_GPa

```
Bond precision:  Fe- N = 0.0055 A        Wavelength=0.28880

Cell:             a=5.4227(6)      b=2.6153(3)      c=10.590(11)
                  alpha=90         beta=90          gamma=90
Temperature:      293 K

                Calculated                Reported
Volume          150.19(16)                150.19(16)
Space group     P n m a                   P n m a
Hall group      -P 2ac 2n                 -P 2ac 2n
Moiety formula  Fe3 N2                    ?
Sum formula     Fe3 N2                    Fe3 N2
Mr              195.57                    195.60
Dx,g cm-3       8.649                     8.648
Z               4                         4
Mu (mm-1)       2.219                     2.056
F000            368.0                     368.0
F000'           369.07
h,k,lmax        11,5,22                   10,5,10
Nref            888                       292
Tmin,Tmax                                 0.490,1.000
Tmin'

Correction method= # Reported T Limits: Tmin=0.490 Tmax=1.000
AbsCorr = MULTI-SCAN

Data completeness= 0.329          Theta(max)= 18.110

R(reflections)= 0.0311( 255)      wR2(reflections)= wR= 0.0344( 292)

S = 1.590                 Npar= 29
```

The following ALERTS were generated. Each ALERT has the format
test-name_ALERT_alert-type_alert-level.
Click on the hyperlinks for more details of the test.

Alert level A

```
PLAT027_ALERT_3_A _diffrn_reflns_theta_full value (too) Low ......       7.77 Degree
```

Author Response: The dataset was incomplete since the data were collected in a diamond anvil cell metallic body of which shadows more than 60% of the reflections.

```
PLAT029_ALERT_3_A _diffrn_measured_fraction_theta_full value Low .      0.520 Note
```

Author Response: The dataset was incomplete since the data were collected in a diamond anvil cell metallic body of which shadows more than 60% of the reflections.

Alert level B

```
PLAT911_ALERT_3_B Missing # FCF Refl Between THmin & STh/L=  0.600         73 Report
```

Author Response: The dataset was incomplete since the data were collected in a diamond anvil cell metallic body of which shadows more than 60% of the reflections.

Alert level C

```
PLAT148_ALERT_3_C s.u. on the      c  - Axis is (Too) Large ......      0.011 Ang.
PLAT790_ALERT_4_C Centre of Gravity not Within Unit Cell: Resd.  #          1 Note
              Fe3 N2
```

Alert level G

```
ABSMU01_ALERT_1_G  Calculation of _exptl_absorpt_correction_mu
                not performed for this radiation type.
PLAT004_ALERT_5_G Polymeric Structure Found with Maximum Dimension          3 Info
PLAT005_ALERT_5_G No Embedded Refinement Details found  in the CIF     Please Do !
PLAT199_ALERT_1_G Reported _cell_measurement_temperature ..... (K)        293 Check
PLAT200_ALERT_1_G Reported   _diffrn_ambient_temperature ..... (K)        293 Check
PLAT804_ALERT_5_G Number of ARU-Code Packing Problem(s) in PLATON          41 Info
PLAT808_ALERT_5_G No Parseable SHELXL Style Weighting Scheme Found     Please Check
PLAT910_ALERT_3_G Missing # of FCF Reflection(s) Below Theta(Min)           1 Note
PLAT912_ALERT_4_G Missing # of FCF Reflections Above STh/L=  0.600        184 Note
PLAT913_ALERT_3_G Missing # of Very Strong Reflections in FCF ....          2 Note
PLAT929_ALERT_5_G No Weight Pars,Obs and Calc R1,wR2,S not checked          ! Info
PLAT952_ALERT_5_G Calculated (ThMax) and CIF-Reported Lmax Differ          12 Units
PLAT958_ALERT_1_G Calculated (ThMax) and Actual (FCF) Lmax Differ          12 Units
```

---

2 **ALERT level A** = Most likely a serious problem - resolve or explain
1 **ALERT level B** = A potentially serious problem, consider carefully
2 **ALERT level C** = Check. Ensure it is not caused by an omission or oversight
13 **ALERT level G** = General information/check it is not something unexpected

```
4 ALERT type 1 CIF construction/syntax error, inconsistent or missing data
0 ALERT type 2 Indicator that the structure model may be wrong or deficient
6 ALERT type 3 Indicator that the structure quality may be low
2 ALERT type 4 Improvement, methodology, query or suggestion
6 ALERT type 5 Informative message, check
```

It is advisable to attempt to resolve as many as possible of the alerts in all categories. Often the minor alerts point to easily fixed oversights, errors and omissions in your CIF or refinement strategy, so attention to these fine details can be worthwhile. In order to resolve some of the more serious problems it may be necessary to carry out additional measurements or structure refinements. However, the purpose of your study may justify the reported deviations and the more serious of these should normally be commented upon in the discussion or experimental section of a paper or in the "special_details" fields of the CIF. checkCIF was carefully designed to identify outliers and unusual parameters, but every test has its limitations and alerts that are not important in a particular case may appear. Conversely, the absence of alerts does not guarantee there are no aspects of the results needing attention. It is up to the individual to critically assess their own results and, if necessary, seek expert advice.

Publication of your CIF in IUCr journals

A basic structural check has been run on your CIF. These basic checks will be run on all CIFs submitted for publication in IUCr journals (*Acta Crystallographica*, *Journal of Applied Crystallography*, *Journal of Synchrotron Radiation*); however, if you intend to submit to *Acta Crystallographica Section C* or *E* or *IUCrData*, you should make sure that full publication checks are run on the final version of your CIF prior to submission.

Publication of your CIF in other journals

Please refer to the *Notes for Authors* of the relevant journal for any special instructions relating to CIF submission.

PLATON version of 27/03/2017; check.def file version of 24/03/2017

Datablock Fe3N2_49.6_GPa - ellipsoid plot

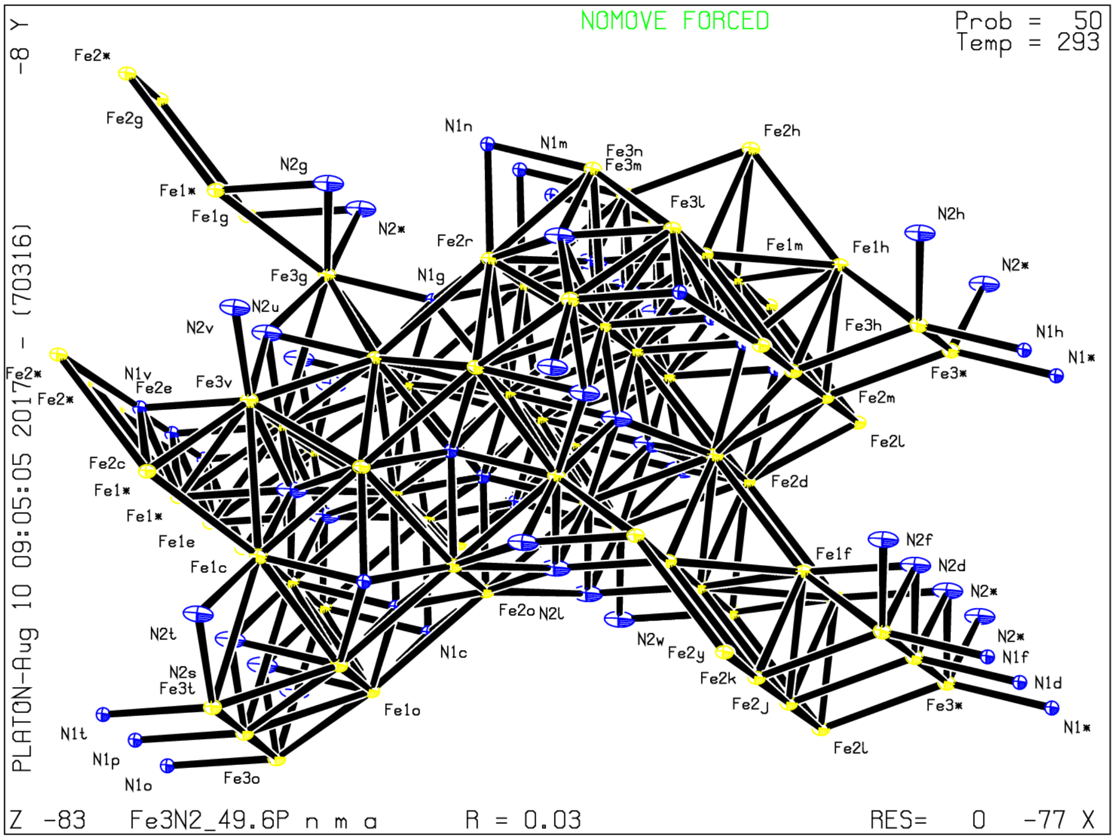

Supplement: Supplementary file 8 — Supplementary Data 5 [file 41467_2018_5143_MOESM8_ESM.pdf]
