# Supplementary material for: Fe-N system at high pressure reveals a compound featuring polymeric nitrogen chains
Source: Nat Commun. 2018 Jul 16;9:2756. doi: 10.1038/s41467-018-05143-2 (PMC6048061; doi:10.1038/s41467-018-05143-2)

checkCIF/PLATON report

Structure factors have been supplied for datablock(s) I

THIS REPORT IS FOR GUIDANCE ONLY. IF USED AS PART OF A REVIEW PROCEDURE FOR PUBLICATION, IT SHOULD NOT REPLACE THE EXPERTISE OF AN EXPERIENCED CRYSTALLOGRAPHIC REFEREE.

No syntax errors found. CIF dictionary Interpreting this report

Datablock: I

```
Bond precision:  = 0.0000 A             Wavelength=0.28880

Cell:                 a=2.62990       b=2.62990       c=4.81870
                      alpha=90        beta=90         gamma=120
Temperature:          293 K

                 Calculated                  Reported
Volume           28.863                      29
Space group      P 63/m m c                  P 63/m m c
Hall group       -P 6c 2c                    -P 6c 2c
Moiety formula   Fe N                        0.08(Fe12 N12)
Sum formula      Fe N                        Fe N
Mr               69.86                       69.85
Dx,g cm-3        8.038                       8.037
Z                2                           2
Mu (mm-1)        1.936                       1.982
F000             66.0                        66.7
F000'            66.17
h,k,lmax         5,5,9                       4,2,5
Nref             67                          37
Tmin,Tmax                                    0.487,1.000
Tmin'

Correction method= # Reported T Limits: Tmin=0.487 Tmax=1.000
AbsCorr = MULTI-SCAN

Data completeness= 0.552          Theta(max)= 17.130

R(reflections)= 0.0638( 35)       wR2(reflections)= 0.1725( 37)

S = 1.112                  Npar= 4
```

The following ALERTS were generated. Each ALERT has the format
test-name_ALERT_alert-type_alert-level.
Click on the hyperlinks for more details of the test.

Alert level A

```
PLAT029_ALERT_3_A _diffrn_measured_fraction_theta_full value Low .      0.544 Why?
```

Author Response: The dataset was incomplete since the data were collected in a diamond anvil cell metallic body of which shadows more than 60% of the reflections.

```
PLAT973_ALERT_2_A Check Calcd Positive Resid. Density on        Fe       3.70 eA-3
```

Author Response: We have used three different refienement programs (Jana2006, Shelxl and olex2.refine) to check this alert. All programs show much smaller residual density on the Fe atom. Therefore, we assume that there s in the checkcif calculation.

Alert level B

```
PLAT911_ALERT_3_B Missing FCF Refl Between Thmin & STh/L=    0.600          6 Report
```

Author Response: The dataset was incomplete since the data were collected in a diamond anvil cell metallic body of which shadows more than 60% of the reflections.

```
PLAT927_ALERT_1_B Reported and Calculated  wR2 Differ by .........    -0.0083 Check
```

Author Response: This difference may be due to the usage of olex2 as the refinement pro

---

Alert level C

```
PLAT088_ALERT_3_C Poor Data / Parameter Ratio ....................       9.25 Note
PLAT141_ALERT_4_C s.u. on a - Axis Small or Missing .............    0.00000 Ang.
PLAT143_ALERT_4_C s.u. on c - Axis Small or Missing .............    0.00000 Ang.
PLAT151_ALERT_1_C No s.u. (esd) Given on Volume ..................     Please Do !
PLAT790_ALERT_4_C Centre of Gravity not Within Unit Cell: Resd.  #          1 Note
              Fe N
PLAT925_ALERT_1_C The Reported and Calculated Rho(max) Differ by .       1.78 eA-3
PLAT926_ALERT_1_C Reported and Calculated   R1 Differ by .........    -0.0012 Check
PLAT971_ALERT_2_C Check Calcd Resid. Dens.  0.73A   From Fe            1.75 eA-3
PLAT972_ALERT_2_C Check Calcd Resid. Dens.  0.39A   From Fe           -2.10 eA-3
PLAT972_ALERT_2_C Check Calcd Resid. Dens.  0.93A   From Fe           -1.56 eA-3
PLAT972_ALERT_2_C Check Calcd Resid. Dens.  0.91A   From Fe           -1.53 eA-3
```

Alert level G

```
FORMU01_ALERT_1_G  There is a discrepancy between the atom counts in the
            _chemical_formula_sum and _chemical_formula_moiety. This is
            usually due to the moiety formula being in the wrong format.
            Atom count from _chemical_formula_sum:   Fe1 N1
```

```
            Atom count from _chemical_formula_moiety:Fe0.96 N0.96
ABSMU01_ALERT_1_G  Calculation of _exptl_absorpt_correction_mu
                not performed for this radiation type.
PLAT004_ALERT_5_G Polymeric Structure Found with Maximum Dimension          3 Info
PLAT042_ALERT_1_G Calc. and Reported MoietyFormula Strings  Differ     Please Check
PLAT072_ALERT_2_G SHELXL First  Parameter in WGHT  Unusually Large       0.14 Report
PLAT199_ALERT_1_G Reported _cell_measurement_temperature ..... (K)        293 Check
PLAT200_ALERT_1_G Reported   _diffrn_ambient_temperature ..... (K)        293 Check
PLAT802_ALERT_4_G CIF Input Record(s) with more than 80 Characters          2 Info
PLAT881_ALERT_1_G No Datum for _diffrn_reflns_av_R_equivalents ...     Please Do !
PLAT910_ALERT_3_G Missing # of FCF Reflection(s) Below Theta(Min).          2 Note
PLAT912_ALERT_4_G Missing # of FCF Reflections Above STh/L=  0.600          5 Note
PLAT913_ALERT_3_G Missing # of Very Strong Reflections in FCF ....          2 Note
PLAT933_ALERT_2_G Number of OMIT Records in Embedded .res File ...          2 Note
PLAT958_ALERT_1_G Calculated (ThMax) and Actual (FCF) Lmax Differ           4 Units
PLAT961_ALERT_5_G Dataset Contains no Negative Intensities .......     Please Check
PLAT984_ALERT_1_G The Fe-f'=       0.349 Deviates from the B&C-Value      0.103   Ch
PLAT984_ALERT_1_G The  N-f'=       0.007 Deviates from the B&C-Value     -0.001   Ch
PLAT985_ALERT_1_G The Fe-f"=       0.853 Deviates from the B&C-Value      0.152   Ch
```

---

  2 **ALERT level A** = Most likely a serious problem - resolve or explain
  2 **ALERT level B** = A potentially serious problem, consider carefully
 11 **ALERT level C** = Check. Ensure it is not caused by an omission or oversight
 18 **ALERT level G** = General information/check it is not something unexpected

```
 14 ALERT type 1 CIF construction/syntax error, inconsistent or missing data
  7 ALERT type 2 Indicator that the structure model may be wrong or deficient
  5 ALERT type 3 Indicator that the structure quality may be low
  5 ALERT type 4 Improvement, methodology, query or suggestion
  2 ALERT type 5 Informative message, check
```

It is advisable to attempt to resolve as many as possible of the alerts in all categories. Often the minor alerts point to easily fixed oversights, errors and omissions in your CIF or refinement strategy, so attention to these fine details can be worthwhile. In order to resolve some of the more serious problems it may be necessary to carry out additional measurements or structure refinements. However, the purpose of your study may justify the reported deviations and the more serious of these should normally be commented upon in the discussion or experimental section of a paper or in the "special_details" fields of the CIF. checkCIF was carefully designed to identify outliers and unusual parameters, but every test has its limitations and alerts that are not important in a particular case may appear. Conversely, the absence of alerts does not guarantee there are no aspects of the results needing attention. It is up to the individual to critically assess their own results and, if necessary, seek expert advice.

Publication of your CIF in IUCr journals

A basic structural check has been run on your CIF. These basic checks will be run on all CIFs submitted for publication in IUCr journals (*Acta Crystallographica*, *Journal of Applied Crystallography*, *Journal of Synchrotron Radiation*); however, if you intend to submit to *Acta Crystallographica Section C* or *E* or *IUCrData*, you should make sure that full publication checks are run on the final version of your CIF prior to submission.

Publication of your CIF in other journals

Please refer to the *Notes for Authors* of the relevant journal for any special instructions relating to CIF submission.

PLATON version of 09/11/2017; check.def file version of 08/11/2017

Datablock I - ellipsoid plot

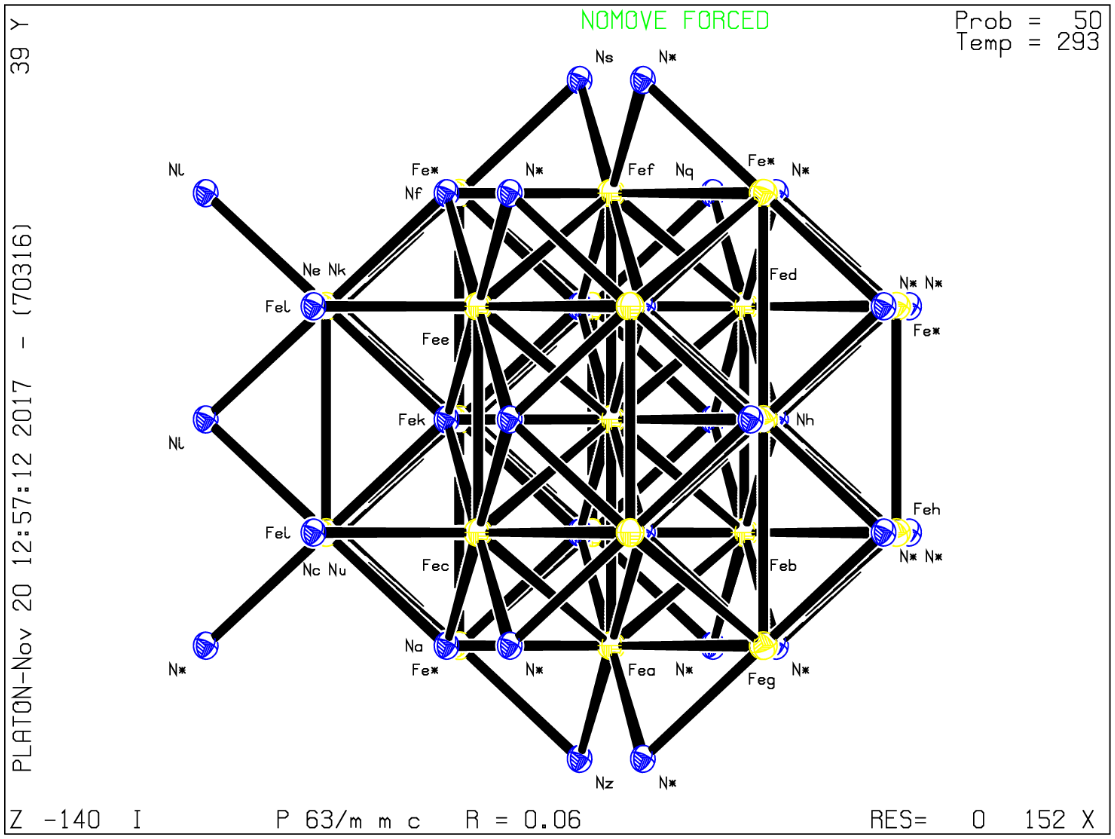

Supplement: Supplementary file 9 — Supplementary Data 6 [file 41467_2018_5143_MOESM9_ESM.pdf]
